# Autologous anti-GD2 CAR T cells efficiently target primary human glioblastoma

**DOI:** 10.1038/s41698-024-00506-z

**Published:** 2024-02-01

**Authors:** Chiara Chiavelli, Malvina Prapa, Giulia Rovesti, Marco Silingardi, Giovanni Neri, Giuseppe Pugliese, Lucia Trudu, Massimiliano Dall’Ora, Giulia Golinelli, Giulia Grisendi, Jonathan Vinet, Marco Bestagno, Carlotta Spano, Roberto Vito Papapietro, Roberta Depenni, Katia Di Emidio, Anna Pasetto, Daniela Nascimento Silva, Alberto Feletti, Silvia Berlucchi, Corrado Iaccarino, Giacomo Pavesi, Massimo Dominici

**Affiliations:** 1 Laboratory of Cellular Therapy, Division of Oncology, Department of Medical and Surgical Sciences for Children & Adults, University of Modena and Reggio Emilia, Modena, Italy; 2 Clinical and Experimental Medicine PhD Program, University of Modena and Reggio Emilia, Modena, Italy; 3 Department of Oncology and Hematology, University-Hospital of Modena and Reggio Emilia, Modena, Italy; 4 Leucid Bio Ltd., Guy’s Hospital, Great Maze Pond, London, SE1 9RT UK; 5 Evotec (Modena) Srl, Medolla, Modena, Italy; 6 Center for Cellular Immunotherapies, Perelman School of Medicine, and Parker Institute for Cancer Immunotherapy at University of Pennsylvania, Philadelphia, PA USA; 7 Centro Interdipartimentale Grandi Strumenti (CIGS), University of Modena and Reggio Emilia, Modena, Italy; 8 International Centre for Genetic Engineering and Biotechnology, Trieste, Italy; 9 Department of Biomedical, Metabolic and Neural Sciences, University of Modena and Reggio Emilia, Modena, Italy; 10 Section for Cell Therapy, Radiumhospitalet, Oslo University Hospital, Oslo, Norway; 11 Department of Laboratory Medicine, Karolinska Institutet, Stockholm, Sweden; 12 Department of Neurosciences, Biomedicine and Movement Sciences, Neurosurgery Unit, University of Verona, Verona, Italy; 13 Department of Biomedical, Metabolic and Neural Sciences, University of Modena and Reggio Emilia - Division of Neurosurgery, Department of Neurosciences, University-Hospital of Modena and Reggio Emilia, Modena, Italy

**Keywords:** CNS cancer, Cancer immunotherapy

## Abstract

Glioblastoma (GBM) remains a deadly tumor. Treatment with chemo-radiotherapy and corticosteroids is known to impair the functionality of lymphocytes, potentially compromising the development of autologous CAR T cell therapies. We here generated pre-clinical investigations of autologous anti-GD2 CAR T cells tested against 2D and 3D models of GBM primary cells. We detected a robust antitumor effect, highlighting the feasibility of developing an autologous anti-GD2 CAR T cell-based therapy for GBM patients.

Glioblastoma multiforme (GBM) is the most frequent and deadly primary glial brain tumor in adults, characterized by a poor response to chemo- and radiotherapy^1^. Autologous chimeric antigen receptor (CAR) T cell therapy has been changing the therapeutic landscape of hematological malignancies^2,3^, and emerging as a promising treatment for solid tumors, including GBM^4,5^. Evidence of treatment feasibility and safety in humans has been shown in clinical studies involving PSMA-targeting CAR T cells against prostate cancer^6^, anti-claudin18.2 CAR T cells in gastrointestinal cancers^7^, and anti-GD2 CAR T cells for diffuse midline glioma (DMG), a peculiar brain tumor occurring in children and young adults^8^.

Regarding GBM, published CAR T cell-based clinical trials have been focused on cell surface targets such as epidermal growth factor receptor vIII (EGFRvIII), human epidermal growth factor receptor 2 (HER2), and IL-13 receptor α chain 2 (IL13Rα2)^4,5^. So far, clinical investigations have exploited the use of autologous CAR T cells based on pre-clinical data obtained from allogeneic settings^5^. The use of pre-clinical models based on allogeneic CAR T cells is often necessary due to lack of sufficient autologous material and this may be particularly true for GBM patients since current treatments often induce severe and long-lasting lymphocyte toxicity^9,10^. In addition, autologous pre-clinical models may be preferable during CAR T development, as the data acquired in these models offer a more robust basis for clinical translation that is currently linked with autologous CAR-T approved for clinical uses.

We and others have previously investigated the efficacy of allogeneic anti-GD2 CAR T cells in pre-clinical models of GBM highlighting a specific antitumor activity, although associated with significant allogeneic background activity^11–13^. In the current study, we tested the antitumor activity of autologous anti-GD2 CAR T cells against 2D and 3D cultures of matched GD2-positive primary tumor cell lines derived from four patients (identified as C6, C10, C12, and C15). Autologous T cells, isolated from cryopreserved PBMCs, were engineered with a vector expressing a second-generation anti-GD2 CAR^11^. The transduction efficiency of these autologous T cells (76.6 ± 14.2%) was comparable to our previous findings^12^ despite the PBMCs freezing/thawing processes, the long cryopreservation interval (up to 37 months) and the previous/current immunosuppressive treatments in three out of four patients included in the study (Table 1). Next to transduction efficiency, the immunophenotype of effector cells derived from two patients has been analyzed (C6 and C15) providing evidence of a mixed population of cytotoxic T lymphocytes (CTL, mostly NKT), CD8^+^ and CD4^+^ with variability among the two donors in terms of lymphocyte subsets (Supplementary Figs. 1 and 2). Despite the differences, autologous anti-GD2 CAR T effectors mediated a specific and robust killing in all conditions (Fig. 1a), except initially (24 h) against the GBM C6 cell line at the lowest E:T ratio (1:5) and at the earliest time point (24 h). The microscopic evaluations confirmed an anti-GD2 CAR mediated anti-GBM effect with the generation of activated lymphocyte clusters appearing overtime at all E:T ratios tested in C6, C10 and C15 patients (Fig. 1b).

**Table 1:** Patients and autologous T cells characteristics.

	Patient ID			
	C6	C10	C12	C15
Diagnosis (WHO 2016)	GBM grade IV	GBM grade IV	GBM grade IV	GBM grade IV
Gender, Age at diagnosis (y)	M, 43	M, 61	M, 77	F, 65
Disease status	Newly diagnosed	Relapsed	Newly diagnosed	Newly diagnosed
Localization (region)	Right temporal	Right temporal	Left temporal	Right occipito-parietal
*MGMT* methylation	Yes	No	No	No
*IDH1* status	wt	wt	wt	wt
*IDH2* status	wt	Not known	Not known	Not known
GD2 expression (%)	92.1	91.5	90.1	89.6
Previous treatments	Concomitant RT-TMZ	Concomitant RT-TMZ and adjuvant TMZ	None	Concomitant RT-TMZ
Timing of lymphocyte collection	Adjuvant treatment (TMZ) - 6th cycle	Surgical resection of relapsed tumor	Surgical resection of newly diagnosed tumor	Adjuvant treatment (TMZ) - 1st cycle
Corticosteroids use at the time of lymphocyte collection	No	Yes	Yes	No
Lymphocyte number (normal range 1000–4500/mmc)	1208/mmc	1350/mmc	n.a.	860/mmc
Lymphocyte use	Frozen	Frozen	Frozen	Frozen
Freezing-Thawing interval	27 months	37 months	36 months	20 months

*ID* identifier, *M* male, *F* female, *y* years, *MGMT* methylguanine methyltransferase, *wt* wild type, *IDH* isocitrate dehydrogenase, *RT* radiotherapy, *TMZ* Temozolomide, *PBMCs* peripheral blood mononuclear cells, *n.a.* not available.

**Fig. 1:**
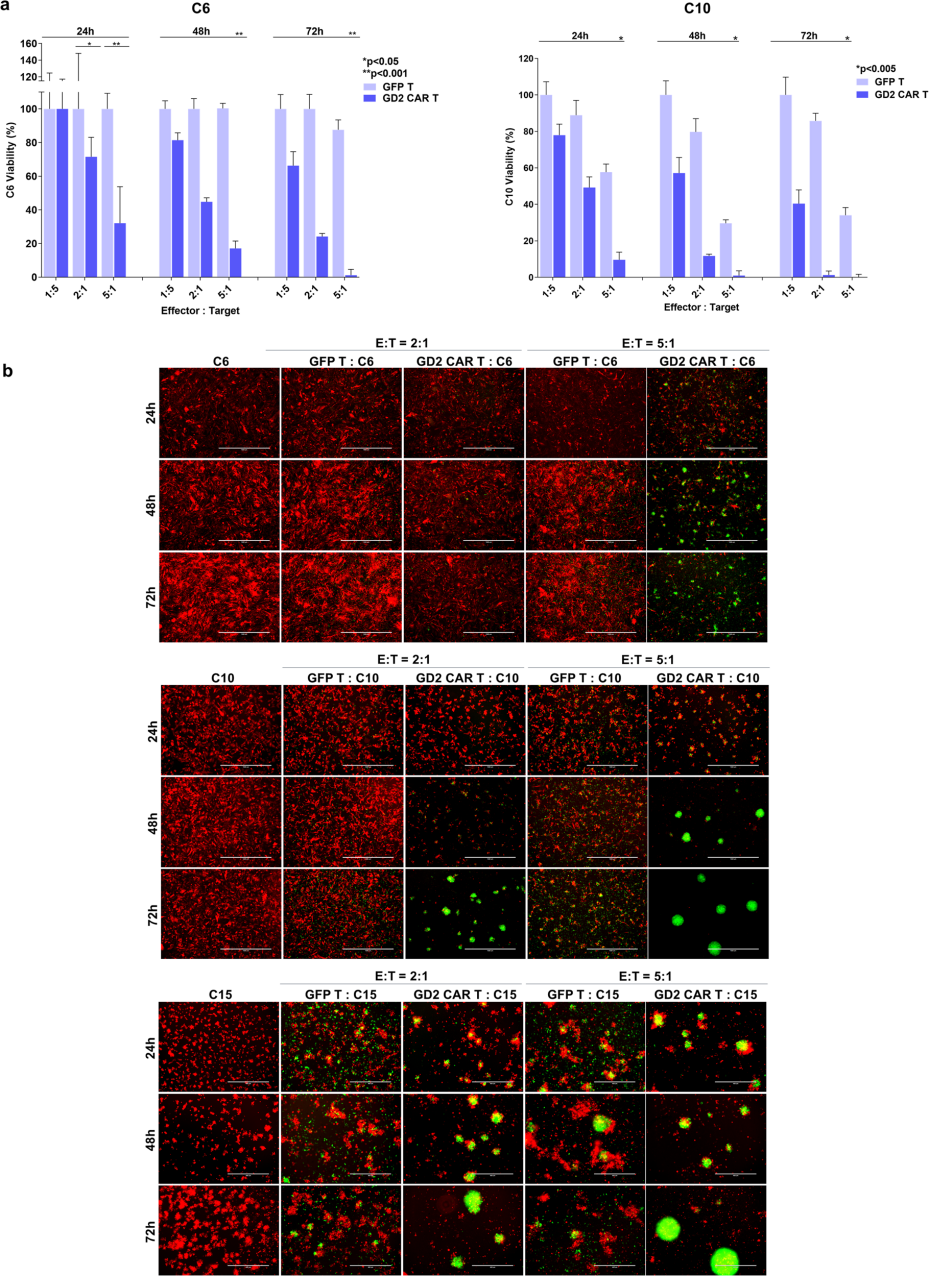
Time- and dose-dependent antitumor activity of autologous anti-GD2 CAR T cells. **a** Patient-derived glioblastoma (GBM) cells (dsRED C6, dsRED C10) are co-cultured with autologous GFP T and anti-GD2 CAR T cells at three different E:T ratios. Tumor viability is evaluated by fluorescence viability assay at three different time points (24, 48, and 72 h). Data are shown as mean ± SD from six technical replicates; *p* values are calculated by unpaired two-tailed t-test. **b** Representative fluorescence micrographs of C6, C10, and C15 GBM cells (red) in co-culture with autologous anti-GD2 CAR T cell (green) and GFP T cells (green) at 2:1 and 5:1 E:T ratio at 24, 48, and 72 h showing a robust activation and cytotoxic activity of autologous anti-GD2 CAR T cells. GFP T cell population does not affect tumor growth. Scale bar: 1000 µm (C6 and C10); 400 µm (C15).

We then compared the anti-GBM activity of autologous and allogeneic cells with or without CAR transduction (Fig. 2a). Cells armored with the CAR construct generated a robust anti-GBM effect at 48 and 72 h, with the allogeneic cells performing better than the autologous for C6 and the autologous CAR T performing better than the allogeneic cells for C10. In all conditions, autologous GFP T cells did not impact tumor viability confirming that the CAR introduction drives a specific and robust anti-GBM effect. To better understand how previously frozen PBMCs perform after transduction and during anti-GBM activity, we evaluated cytokine release during cytotoxicity assay shown in Fig. 1b for C15. Thus, we compared autologous CAR T cells from C15 patient, generated after 20 months of storage, with freshly generated allogeneic CAR T cells derived from a healthy donor. We collected supernatants from co-cultures of both autologous and allogeneic effectors against GBM C15 target cells at three different time points (24, 48, and 72 h) and analyzed 7 different cytokines: IL-10, IL-2, Granzyme B, Granzyme A, IFNγ, TNFα, and perforin. The results show that autologous anti-GD2 CAR T cells have a comparable, if not even higher, cytokine release than allogeneic CAR T cells (Fig. 2b).

**Fig. 2:**
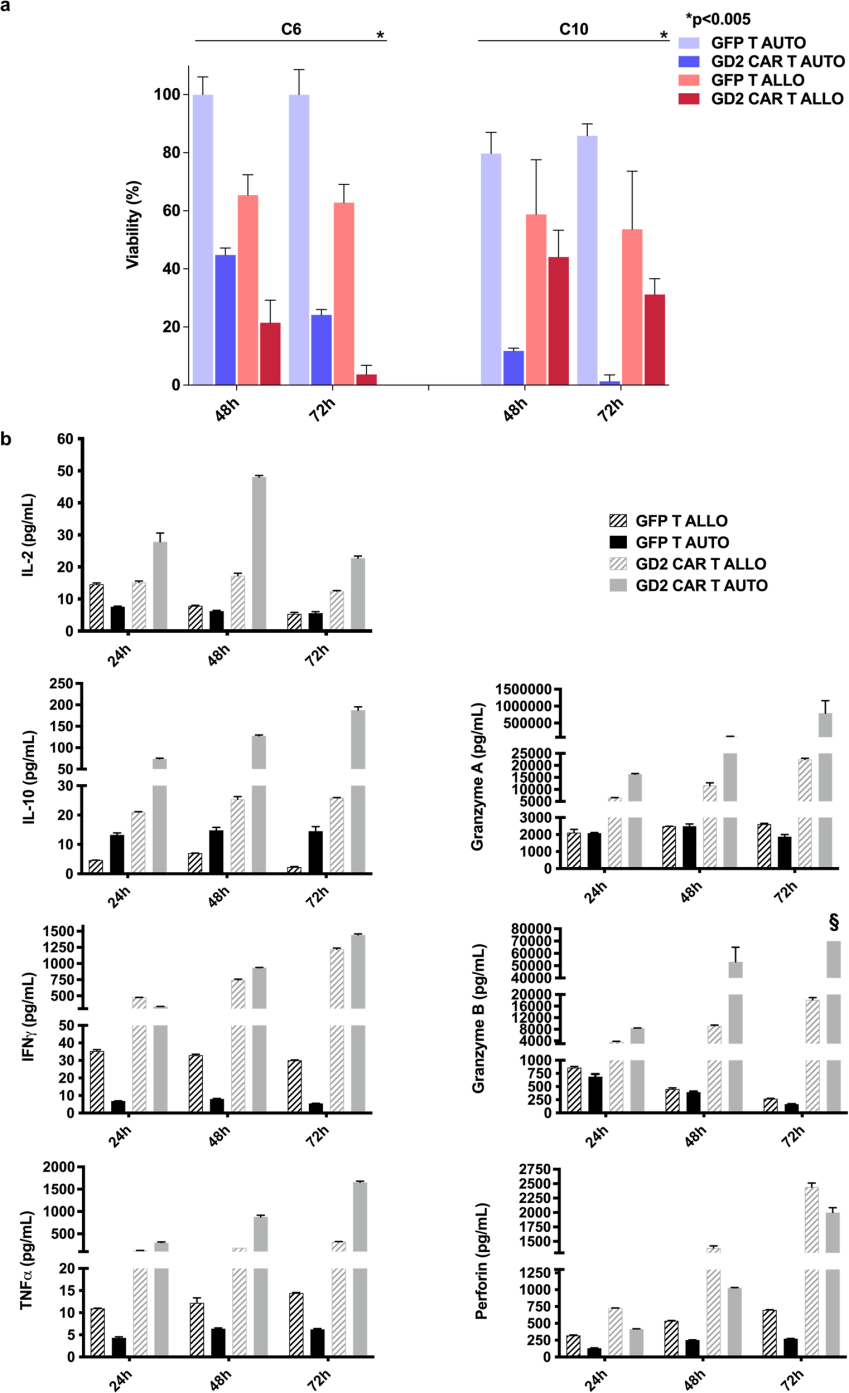
Killing effect and cytokine release comparison between autologous and allogeneic settings in 2D in vitro GBM models. **a** Patient-derived dsRED-labeled glioblastoma (GBM) lines C6 and C10 are co-cultured with either autologous (AUTO) or allogeneic (ALLO) GFP T and anti-GD2 CAR T cells at 2:1 E:T ratio. Tumor viability is evaluated by fluorescence assay at 48 and 72 h. Data are shown as mean ± SD from three technical replicates; *p* values are calculated by unpaired two-tailed t-test. **b** Cytokine release profile comparing previously frozen autologous versus allogeneic effector cells in co-culture with GBM C15 cells. Supernatants of co-culture at 2:1 E:T ratio are assessed for cytokine release at 24, 48, and 72 h. Cytokine level is measured by Simple Plex ELLA (Bio-Techne), and the concentration is reported as pg/mL. §: out of range.

The antitumor effect of anti-GD2 CAR T cells against GBM cells of C6, C10, and C12 patients was then additionally evaluated in 3D spheroid models to further challenge the observed anti-GBM activity (Fig. 3). This approach was established accounting for (i) the more aggressive features of cells growing in 3D, (ii) the complexity of GBM growth, (iii) the heterogeneity of cell subsets in spheres with different levels of apoptosis resistance, (iv) the known immunosuppressive GBM microenvironment in 3D, and (v) the limits of CAR T penetration in solid tumors^14–16^. Confirming our previously reported data^12^, allogeneic anti-GD2 CAR T counteracted primary human GBM spheroids growth. In the autologous settings, GFP T lymphocytes surrounded the spheroids without penetrating the GBM spheres at all time points. On the contrary, the presence of the CAR allowed autologous T cells to enter and disrupt GBM spheroids starting from 24 h (Fig. 3).

**Fig. 3:**
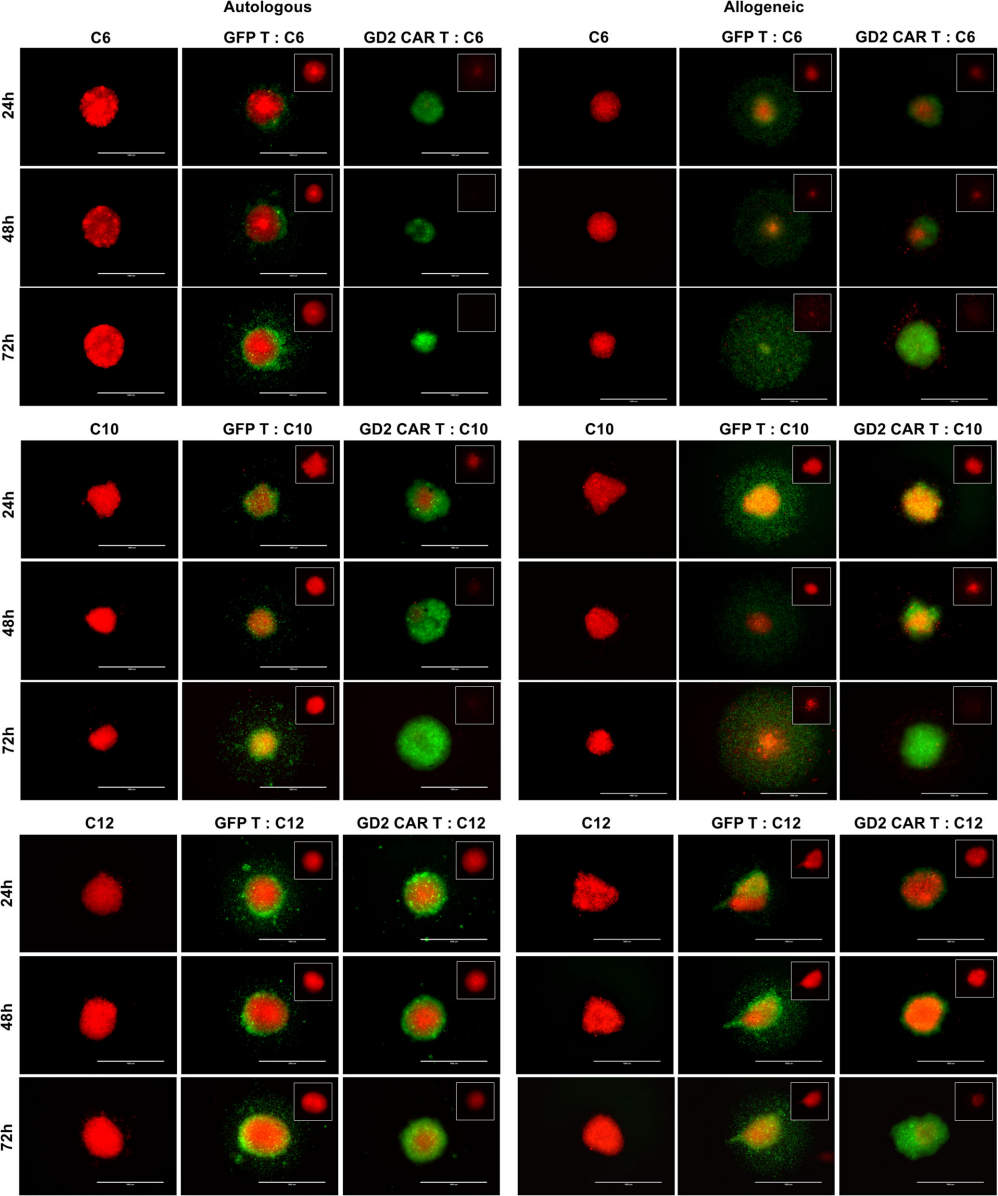
Comparison of autologous versus allogeneic CAR T cells and controls targeting GBM spheroids. Representative fluorescence microscopy images of 3D spheroid assays from C6, C10, and C12 patients. In the first column of each panel, GBM spheroids alone are showed; in the second and the third column the co-cultures at 2:1 E:T ratio with both (autologous or allogeneic) GFP T and anti-GD2 CAR T cells, respectively, are showed. Antitumor activity is monitored at 24, 48, and 72 h. Tumor cells in red, anti-GD2 CAR T cells and GFP T cells in green. Scale bar: 1000 µm.

Aiming at reinforcing our approach and getting closer to a more complex tumor microenvironment, we then generated a 3D spheroid co-culture of the C15 GBM line with either allogeneic or autologous CAR T cells with and without healthy microglial cells (HMC3 cell line with a GD2 expression of 10.4%, not shown). Microglia represent a relevant cell type of the brain playing a central role in the functioning of healthy brain tissue, being also associated with the establishment of an immunosuppressed niche in GBM^17^. Therefore, we considered microglia relevant for a CAR T cell-based strategy, not only for their possible inhibitory action but also for the potential risk of off-target effects due to the reported variable levels of GD2 in glial cells^18^. First, we verified whether C15 cells alone could generate GBM spheroids (Fig. 4a, upper-left pictures), then we successfully targeted them by either autologous or allogeneic CAR T lymphocytes (and controls; Fig. 4a, first and second row), confirming how GBM spheroids can be tackled by autologous CAR T cells. Afterward, we combined the C15 GBM cells (red) with HMC3 microglia cells (purple) with the evidence of a more compact structure (Fig. 4a, third and fourth row). These more complex spheroids were inefficiently penetrated by GFP T cells, despite their intra-GBM clusters. On the contrary, anti-GD2 CAR T cells were able to efficiently tackle GBM cells with their apparent complete eradication, suggesting that microglial cells do not abrogate the activity of both allogeneic and autologous anti-GD2 CAR T cells (Fig. 4a, third and fifth columns). To further confirm these findings, we approached the assay using Luciferase-labeled C12 cells combined with HMC3 microglia cells monitored by luminescence assay to better quantify the data in an additional GBM line (Supplementary Fig. 3). Even in this model and starting from 48 h of co-culture with autologous anti-GD2 CAR T, we were able to significantly reduce GBM viability even in the presence of glial cells (Supplementary Fig. 3). Moreover, taking advantage of our previous experience in testing cell-based approaches in 3D in vitro cultures^19,20^, we generated a GBM model where glial cells (purple) and two GBM primary cell lines (C6 and C12 in red) were respectively loaded into scaffold-based bioreactors (Fig. 4b, left and right panels). Once red and purple cells were engrafted into the 3D matrix (Fig. 4b, first and fourth columns), the systems were perfused by autologous anti-GD2 CAR T cells (and controls) generating a robust cytotoxic activity against GBM with the reduction of red signals even in the presence of microglia (Fig. 4b, third and sixth columns), once again confirming the therapeutic profile of our strategy against GBM.

**Fig. 4:**
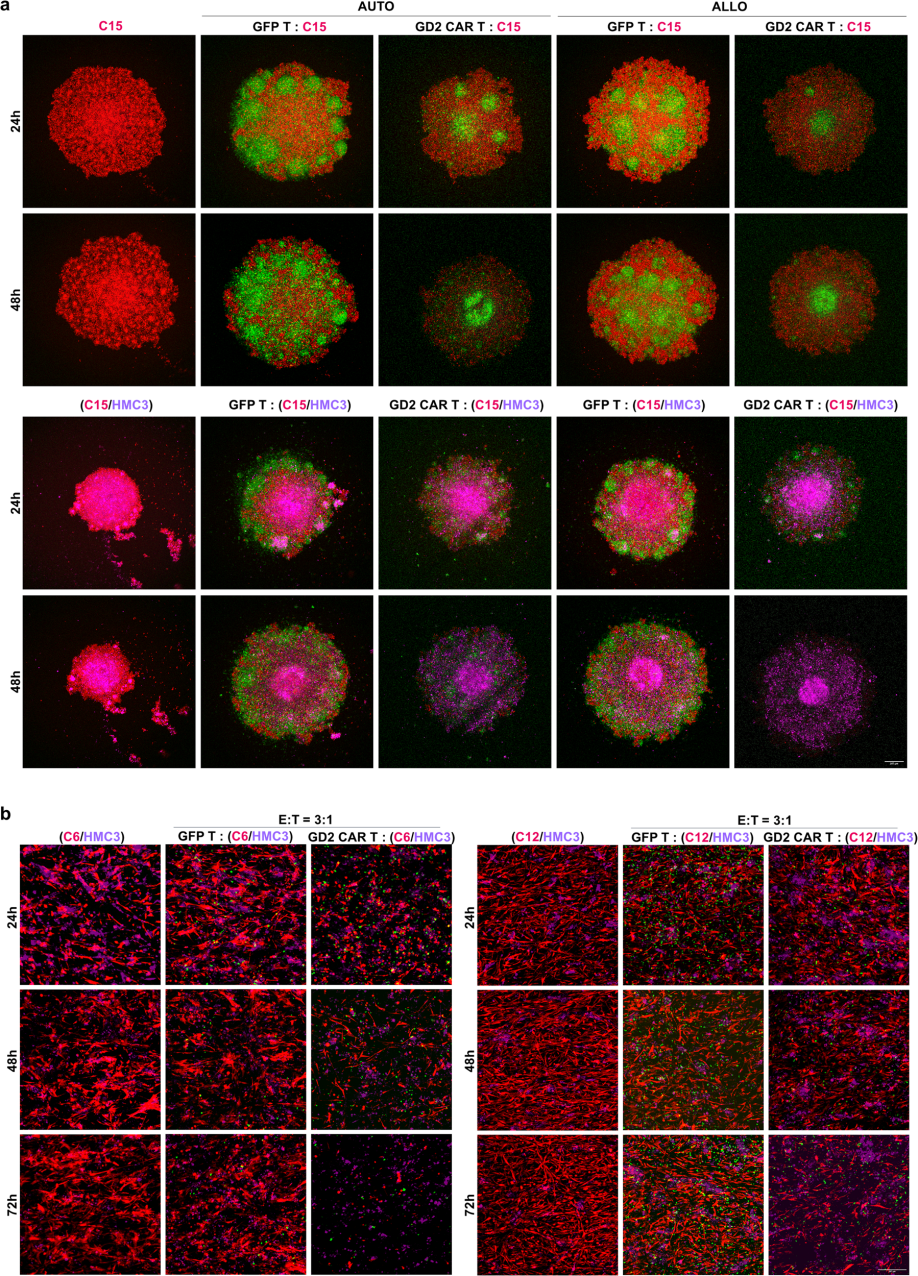
Three-dimensional in vitro models evaluating the impact of healthy microglia on anti-GD2 CAR T antitumor activity. **a** Representative fluorescence microscopy images of GBM C15 spheroids (red) with or without healthy microglia (HMC3 in purple), in co-culture with either autologous or allogeneic anti-GD2 CAR T or GFP T cells (green) at 2:1 E:T ratio. CAR T cells antitumor activity is monitored at 24 and 48 h. Scale bar: 257 μm. **b** Representative fluorescence microscopy images of dsRED C6 and C12 cells with HMC3 cells, forming a GBM-like structure in the VITVO® 3D bioreactor at 24, 48, and 72 h and then in co-culture with autologous anti-GD2 CAR T and GFP T cells at 3:1 E:T ratio. C6 and C12 cells in red, anti-GD2 CAR T cells and GFP control T cells in green, HMC3 in purple. Scale bar: 257 μm.

Reports on pre-clinical models utilizing matching autologous CAR T cells and primary tumor cell lines are uncommon^21^. This is mostly due to the difficulties in obtaining primary cancer cells and enough circulating lymphocytes to enable the generation of CAR T cells^9,10^, in particular from peripheral blood of chemotherapy/immunosuppressive therapy-treated GBM subjects. Our study represented a unique opportunity to evaluate the feasibility and potency of autologous CAR T for GBM, detecting antitumor activity in co-cultures by challenging 3D models, recently considered relevant for clinical translation by regulatory bodies^22^.

Despite the success, autologous CAR T cells have some drawbacks such as costs, production failure, and risk of treatment delay^23^. For these reasons, researchers have been starting to explore the possibility of manufacturing off-the-shelf allogeneic CAR T cells with several technological approaches. Although very promising, allogeneic CAR T cells may, in turn, be associated with major concerns: graft-versus-host disease (GVHD) and risk of limited antitumor activity because of rapid elimination of infused cells by the host immune system^24^. In addition, the use of allogeneic CAR T cells may not be ideal for brain cancer for the risk of allogeneic response causing CNS toxicity, a known side effect of CAR T therapies^25^. Thus, while research in this field is very active and off-the-shelf approaches might gain relevant space in the future, we presume autologous CAR T cells may still be very relevant in selected solid tumor types, such as GBM.

In this context, a sufficient number of T lymphocytes will be an essential requirement for successful CAR T manufacturing^26^ as well as the identification of factors that may affect characteristics of a leukapheresis product, as conceived for hematological diseases^27^. A basic requirement is the collection of a sufficient number of T lymphocytes, frequently impaired by previous/concurrent medications. In our study, we managed to produce active CAR T cells from PBMCs isolated (from 20 to 30 ml of peripheral blood) despite a relatively low number of lymphocytes at the time of blood collection. We believe that this is a relevant achievement in a pre-clinical setting. Certainly, when moving to the clinical scenario, it will be important to carefully consider the timing of PBMCs collection by leukapheresis to provide enough cells as a pre-requisite of a new therapeutic approach in GBM.

Indeed, standard treatments for both newly diagnosed and relapsed GBM patients struggle to provide a substantial survival benefit and new approaches are eagerly demanded. Our data, showing that autologous anti-GD2 CAR T cells mediated a powerful and specific antitumor response against matched human primary GBM cells, encourage the development of a CAR T-based therapeutic option for GBM indicating how this approach may be close to a possible clinical implementation.

## Methods

### Primary glioblastoma cell isolation, culture, and transduction

Primary GBM samples and peripheral blood were obtained from patients affected by high-grade glioma who underwent surgery at the Neurosurgery Unit (NOCSAE, Baggiovara, University Hospital of Modena). Samples were collected after signed informed consent from patients. The study was performed following the ethical standards as laid down in the Declaration of Helsinki and its later amendments. It was validated by the Ethical and Institutional Review Board at the University Hospital of Modena (prot. N.3600/C.E. September 2017). Tumor samples were dissociated and GBM cell lines were isolated as described^12^. Once cells started forming spheroids, the medium was replaced every 2 to 3 days, and the reseeding density was 20,000 cells/cm^2^. Primary tumor samples (ID: C6, C10, C12, and C15) were transduced by viral particles encoding for dsRED, as published^12^, or transiently marked with a fluorescent probe (for C15; CellTracker™ Orange CMRA; Thermo Fisher Scientific, Massachusetts, USA; #C34551). GBM primary cell lines were tested routinely for the absence of mycoplasma (MycoAlert Mycoplasma Detection Kit and MycoAlert Control Set, Lonza, Basel, Switzerland; #LOLT07318, #LOLT07518).

### Healthy microglia culture

HMC3 cells (ATCC® #CRL-3304™) were cultured as recommended by ATCC. The base medium for this cell line is EMEM (ATCC® #30-2003™) supplemented with 10% heat-inactivated defined fetal bovine serum (FBS; 500 ml, Gibco, #10270-106, and 1% Penicillin/Streptomycin (Pen/Strep; 100 ml, Gibco, #15140-122). HMC3 cells were transiently marked with a fluorescent probe (CellTracker™ Deep Red; Thermo Fisher Scientific, Massachusetts, USA; #C34565).

### CAR T cells manufacturing

CAR T cells were engineered starting from GBM patients’ PBMCs (as approved by the Institutional Review Board) which were separated by a density gradient (Lymphoprep, Alere Technologies AS, Oslo, Norway; #1114545) and then plated in RPMI 1640 (Gibco; #52400-017) with 1% FBS, 1% glutamine and 1% penicillin-streptomycin. Non-adherent cells were pre-stimulated for 48 h in 7 µg/mL Phytohemagglutinin (PHA-M, Sigma-Aldrich; #L8902) at a concentration of 1 × 10^6^ cells/mL in a culture medium made by RPMI 1640 supplemented with 10% heat-inactivated defined FBS (HyClone Laboratories, Utah, USA; #SH30070.03), 500 UI/mL recombinant human Interleukin-2 (rhIL-2, Proleukin, Clinigen Healthcare Ltd, Staffordshire DE14 2WW, UK; #801313AY). Isolated T lymphocytes were transduced by a retroviral vector bearing second-generation anti-GD2 CAR and GFP control vector^11,12^.

### Flow cytometry

The expression of GD2 antigen on isolated tumor cells was assessed by FACS analysis. Primary unconjugated mouse antihuman GD2 (BD, Franklin Lakes NJ, USA; #554272, 1:20) and a secondary APC-conjugated goat anti-mouse Ig (APC Goat Anti-Mouse Ig polyclonal multiple adsorptions; BD; #550826, 1:20) were used for staining. FACS analyses were also performed on anti-GD2 CAR T and GFP T cells manufactured from selected PBMCs of two GBM patients (C6 and C15). Three panels of antibodies were used to detect cell subsets, for cytotoxic T lymphocyte (CTL) labeling: iTCR/TCR γ/δ (APC, Biolegend, #331212), CD56 (PE, BD, #345812), CD3 (PerCP, BD, #345766), CD8 (PE-Cy7, Biolegend, #344750); for memory T lymphocyte labeling CD45RA (APC, Biolegend, #304112), CD3 (FITC, BD, #349201), CD4 (PE, Biolegend, #345769), CCR7 (PerCP, Biolegend, #353219), CD8 (PE-Cy7). Gating strategy is reported in Supplementary Fig. 2. Dilution of all the antibodies 1:20. The transduction efficiency of anti-GD2 CAR T and GFP T cells was determined by the percentage of GFP-positive cells. Data were collected by BD FACSAria III (BD) and analyzed using BD FACSDiva software (BD).

### Cytotoxicity assay in 2D

Autologous anti-GD2 CAR T or GFP T cells were used as effectors (E) and human GD2-positive primary cell lines as targets (T). Different effector-to-target (E:T) ratios were used to assess the cell viability using 96-well black plates (Corning, Kennebuck, ME, USA; #3904) and GloMax Discover Multimode as microplate reader (Promega, Madison, WI, USA). Cultures containing the medium alone or 1% Triton X-100 were used as controls, representing 100% and 0% cell viability, respectively. Average viability was calculated as 100 × (experimental fluorescence − 0% viability fluorescence)/(100% viability fluorescence − 0% viability fluorescence). Co-cultures were also monitored by EVOS FL auto (Thermo Fisher Scientific) over 72 h.

### ELISA

Cytokines in supernatants of cell co-cultures were assessed by automated immunoassay workflow. In detail, IL-2, IL-10, IFNγ, TNFα, Granzyme A, Granzyme B, and perforin were quantified by the automated ELISA platform “Simple Plex ELLA” (ProteinSimple, Bio-Techne, Minneapolis, MN, USA). Instrument calibration was performed using the in-cartridge factory standard curve, and supernatant samples were diluted 1:2 in Sample Diluent (ProteinSimple).

### Cytotoxicity assays in 3D models

In vitro 3D cultures were performed through the formation of spheres. dsRED-positive GBM C6, C10, and C12 spheres were obtained by seeding 20,000 cells/well in a 96-well plate (Sphera Low-Attachment Surface, ThermoFisher; #174927). After 24 h, GBM spheres were co-cultured with transduced lymphocytes at an E:T ratio of 2:1 over 3 days. Co-cultures were also monitored by EVOS FL auto (Thermo Fisher Scientific) over 72 h. C15 cells transiently marked with a red fluorescent probe were seeded at 30,000 cells/well to form spheroids with or without microglia cells (HMC3) at 15,000 cells/well. HMC3 cells were transiently marked with a different fluorescent probe (CellTracker™ Deep Red). These spheroids were then co-cultured with transduced lymphocytes at an E:T ratio of 2:1 over 72 h. Representative images were collected by Leica SP8 confocal microscope (Leica Microsystems GmbH, Wetzlar, Germany). In addition, a 3D bioreactor (VITVO®, EIR Biotherapies srl, Mirandola, Italy; #F000001) was used to evaluate the cytotoxic effect on target cells mediated by CAR T cells at an E:T ratio of 3:1 from 24 h to 72 h. The bioreactor was loaded with 5.0 × 10^5^ dsRED C6 or C12 cells in combination with 2.5 × 10^5^ Deep Red HMC3 cells in 1.4 mL of culture media by a 3 mL syringe (Becton Dickinson and Co, Franklin Lakes, NJ, USA). After 24 h, effector T cells were added. Representative fluorescence images of VITVO® were collected by Nikon A1R confocal microscope (Nikon Instruments Inc., Melville, NY, USA). Quantification of antitumor activity exerted by anti-GD2 CAR T cells and control GFP T cells was obtained seeding composite spheroids with C12Luc GBM cells and HMC3 cells, in co-culture with lymphocytes. We used GloMax Discover Multimode as microplate reader (Promega, Madison, WI, USA). Cultures containing the medium alone or 1% Triton X-100 were used as controls, representing 100% and 0% cell viability, respectively. Average viability was calculated as 100 × (experimental fluorescence − 0% viability fluorescence)/(100% viability fluorescence − 0% viability fluorescence).

### Statistical analysis

GraphPad Prism version 6.0 (GraphPad Prism software, San Diego, CA, USA) was used to generate graphs and perform statistical analysis. In vitro data are expressed by means ± standard deviation (SD). To determine statistical significance, unpaired two-tailed Student’s t-test was used to determine statistical significance. A level of *p* value < 0.05 was used to designate significant differences.

### Reporting summary

Further information on research design is available in the Nature Research Reporting Summary linked to this article.

### Supplementary information

Supplementary Information

Reporting Summary

## Data Availability

All data supporting the findings of this study are available within the paper and its Supplementary Information.
