# Supplementary material for: Autologous anti-GD2 CAR T cells efficiently target primary human glioblastoma
Source: NPJ Precis Oncol. 2024 Feb 1;8:26. doi: 10.1038/s41698-024-00506-z (PMC10834575; doi:10.1038/s41698-024-00506-z)
Supplement: Supplementary file 1 — Supplementary Information [file 41698_2024_506_MOESM1_ESM.pdf]

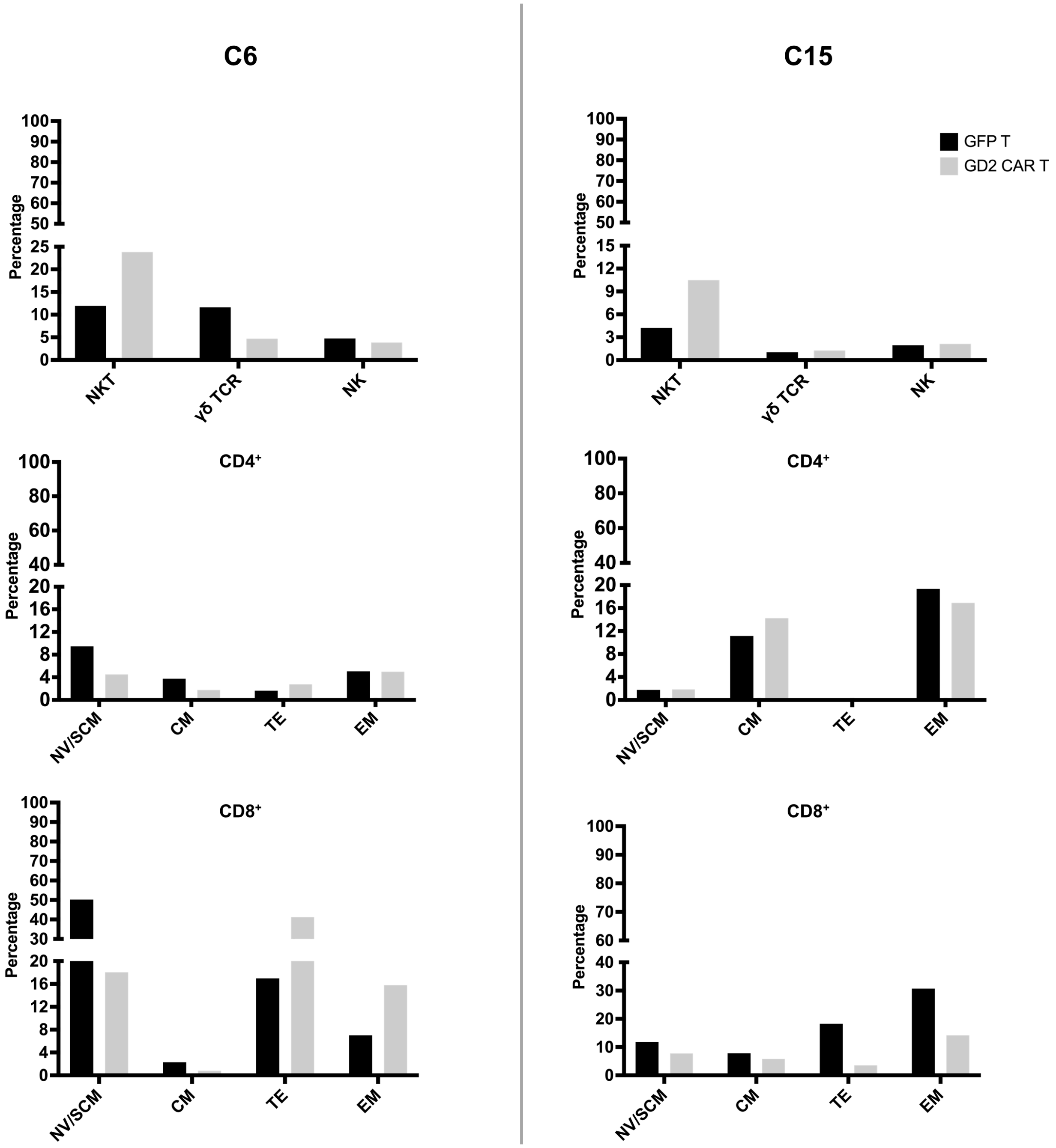

Supplementary Figure 1. Immunophenotype of autologous effector cells. Immunophenotype of GFP T and anti-GD2 CAR T cells generated from PBMCs of two GBM patients C6 and C15. Cytotoxic T Lymphocyte panel: percentages of γδ T (morphological/$CD3^+$/TCR $γδ^+$), NKT (morphological/$CD3^+$/$CD56^+$) and NK cells (morphological/$CD3^-$/$CD56^+$) (in the first row). Memory panel: percentages of N/SCM ($CCR7^+$/$CD45RA^+$), CM ($CCR7^+$/$CD45RA^-$), TE ($CCR7^-$/$CD45RA^+$) and EM ($CCR7^-$/$CD45RA^-$) cells within the $CD4^+$ (morphological/$CD3^+$/$CD4^+$) and $CD8^+$ (morphological/$CD3^+$/$CD8^+$) subpopulations (second and third row, respectively). Abbreviations: N/SCM, naïve/stem cell memory; CM, central memory; TE, effector; EM, effector memory.

Cytotoxic T Lymphocyte panel:
NKT, $\gamma\delta$, NK

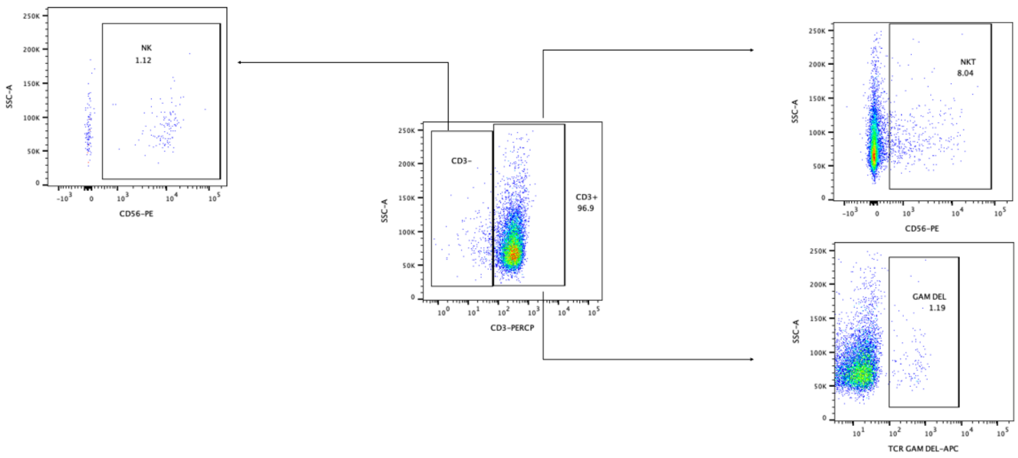

Memory panel:
N/SCM, CM, TE, EM

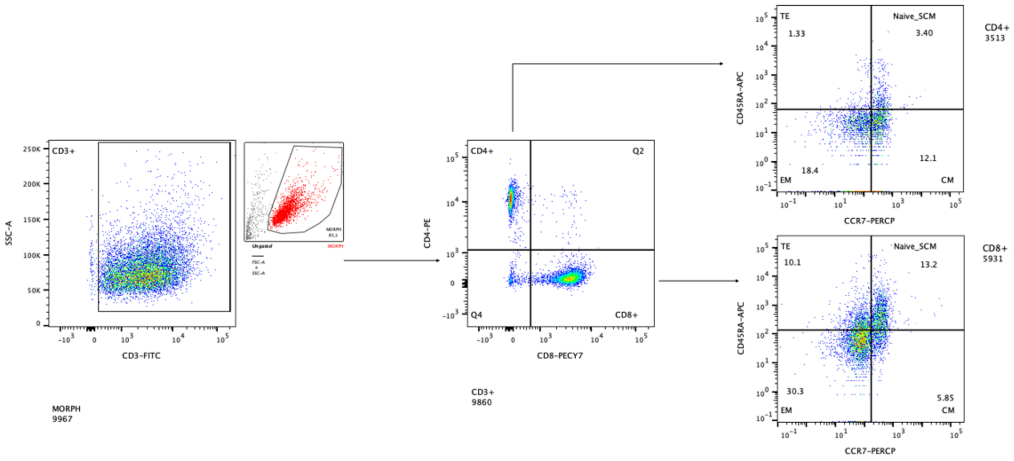

Isotype control panel

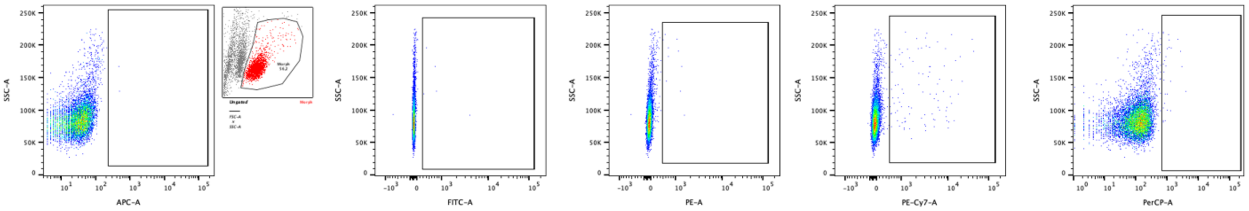

Supplementary Figure 2. Representative gating strategy for effector cells. All the data collected by flow cytometry refer to viable cells gated on the total number of cells in the samples. Gating strategy example for CD4^{+} central memory cells: alive cells/CD3^{+}/CD4^{+}/CCR7^{+}/CD45RA^{-}. Positivity is based on isotype controls. Data are related to C15 GFP T cells.

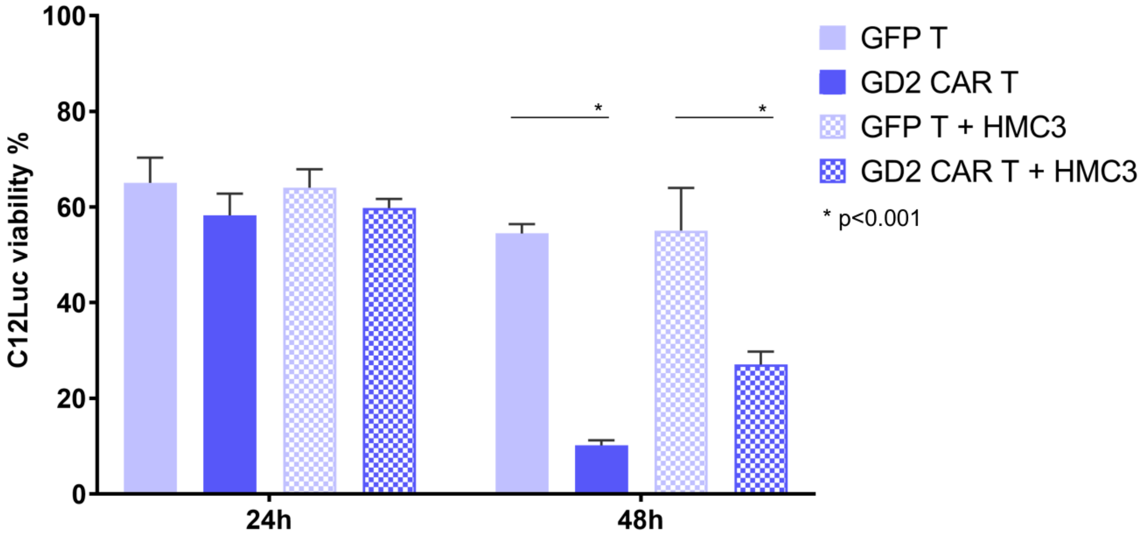

Supplementary Figure 3. Evaluation of antitumor activity in 3D composite spheroid models in luciferase labelled GBM cells. Antitumor activity exerted by anti-GD2 CAR T cells and control GFP T cells was obtained by seeding composite spheroids with GBM C12Luc and HMC3 cells, in co-culture with lymphocytes. Tumor viability is evaluated by luminescence assay at 24h and 48h. Data are shown as mean ± SD from four technical replicates; p values are calculated by unpaired two-tailed t-test.
